# Lignin Degradation Efficiency of Chemical Pre-Treatments on Banana Rachis Destined to Bioethanol Production

**DOI:** 10.3390/biom8040141

**Published:** 2018-11-09

**Authors:** Stefania Costa, Irene Rugiero, Christian Larenas Uria, Paola Pedrini, Elena Tamburini

**Affiliations:** 1 Department of Life Science and Biotechnology, University of Ferrara, Via L. Borsari 46, 44121 Ferrara, Italy; stefania.costa@unife.it (S.C.); irene.rugiero@unife.it (I.R.); pdp@unife.it (P.P.); 2 Laboratorio de Biotecnología, Universidad Poilitécnica Salesiana, Av. Isabel La Católica N 23-52 y Madrid, Quito-Ecuador 170109, Quito, Ecuador; clarenas@ups.edu.ec

**Keywords:** delignification, *organosolv*, oxidation, hypochlorous acid, rachis, lignocellulosic materials, Fourier transform infrared spectra

## Abstract

Valuable biomass conversion processes are highly dependent on the use of effective pretreatments for lignocellulose degradation and enzymes for saccharification. Among the nowadays available treatments, chemical delignification represents a promising alternative to physical-mechanical treatments. Banana is one of the most important fruit crops around the world. After harvesting, it generates large amounts of rachis, a lignocellulosic residue, that could be used for second generation ethanol production, via saccharification and fermentation. In the present study, eight chemical pretreatments for lignin degradation (*organosolv* based on organic solvents, sodium hypochlorite, hypochlorous acid, hydrogen peroxide, alkaline hydrogen peroxide, and some combinations thereof) have been tested on banana rachis and the effects evaluated in terms of lignin removal, material losses, and chemical composition of pretreated material. Pretreatment based on lignin oxidation have demonstrated to reach the highest delignification yield, also in terms of monosaccharides recovery. In fact, all the delignified samples were then saccharified with enzymes (cellulase and beta-glucosidase) and hydrolysis efficiency was evaluated in terms of final sugars recovery before fermentation. Analysis of Fourier transform infrared spectra (FTIR) has been carried out on treated samples, in order to better understand the structural effects of delignification on lignocellulose. Active chlorine oxidations, hypochlorous acid in particular, were the best effective for lignin removal obtaining in the meanwhile the most promising cellulose-to-glucose conversion.

## 1. Introduction

During the last years, shortages of petroleum-based energy, fast resources depletion and increasing problem of CO_2_ emissions have arisen the interest for alternative fuels and more sustainable energy supply in many countries [1]. Among the so-called clean energies, ethanol is considered particularly promising because of some known advantages such as clean burning characteristics, reduction of particulate and NO_x_ emission from combustion, and so on [2]. However, as it is well recognized, recently it is not sustainable to produce ethanol from bioconversion of starchy materials especially because it competes with food and feed chains [3]. Therefore, its production from non-grain feedstock as lignocellulosic biomass has been becoming a hot spot in many countries, due to their eco-friendly nature and low cost availability [4]. Lignocellulosic biomass, with a worldwide production estimated in 200 × 10^9^ tons/year is rightly so considered as the only foreseeable, feasible and sustainable raw material for biofuel production [5] and for the ultimate consolidation of the *biorefinery* concept [6].

Various technological developments based on saccharification and fermentation have improved for the conversion of these substrates into bioethanol [7]. Agricultural residues, food processing wastes, and forestry residues are all potential sources of fermentable sugars to be converted in bioethanol, but the typical recalcitrance of lignocellulosic biomass to enzyme and microorganisms’ attack necessitates of pretreatment as unavoidable pre-requisite [8]. The main focus of those pretreatments is indeed to remove from the plant cell wall the barrier due to lignin, pectin, hemicellulose, glucans, and their spatial interlinks, for increasing the enzymatic and microbial digestibility of cellulose [9].

Different pretreatment technologies have been already extensively described in terms of the mechanisms involved, advantages and disadvantages, and economic assessment [10]. They include biological [11], physical-mechanical [12], chemical [13] methods, and various combinations thereof [14]. 

Even though a biological approach would be in perspective the ideal solution, because it is less harmful to the environment and needs milder conditions, it still necessitates improvements in process duration and cost reduction, factors that strongly limit their actual efficient industrial application [15,16]. To date, chemical pretreatments are the best alternative to steam explosion, because they are more effective and enhance the biodegradation of complex and particularly recalcitrance woody materials as lignin and xylan, recently becoming potentially sustainable in terms of costs and hazardous waste thanks to solvents recovery and recycling [17,18,19]. Moreover, oxidation formed less toxic furan aldehydes and permits a better enzymatic convertibility of cellulose than for the steam-exploded material [20]. To this group belong all pretreatments that involved chemical reactions for the lignocellulosic structure disruption by means of organic or inorganic acids [21], alkali [22], organic solvents (*organosolv*) [23], and oxidative reagents as ozone [24] or active chlorine [25]. Recent developments have indicated that the effects of biological combined with oxidation pretreatment permits to improve the efficiency of delignification and enzymatic saccharification [26]. 

After delignification, residual biomass must be hydrolyzed to produce glucose which is then converted to bioethanol by *Saccharomices cerevisiae* or other microorganisms via alcoholic fermentation [27]. Even though concentrated acid hydrolysis process has a long history of commercial use [28], hydrolysis can be also accomplished by fungal and bacterial hydrolyzing enzymes [29]. There are several enzymes which are required for complete hydrolysis of biomass such as cellulase, xylanase, ligninase, pectinase, etc., among which cellulase is the most important one. Cellulase is a multi-enzyme complex of three different enzymes, exoglucanase, endoglucanase, and beta-glucosidase which acts synergistically for complete hydrolysis of cellulose to cellobiose (an intermediate product of cellulose hydrolysis) and finally to glucose [30].

In the present study, eight chemical pretreatments and some combinations thereof for lignin degradation have been tested on lignocellulosic feedstock as banana rachis and the effects evaluated. 

Treatments with different mechanism have been chosen: with organic solvents (*organosolv*) which remove lignin and hemicellulose by solubilization and extraction [31], with strong oxidants as active chlorine species by demethylation and aromatic substitution [32] and hydrogen peroxide by reaction among radicals and phenolic structure of lignin [33]. The pretreated samples were then hydrolyzed with cellulase enzymes pool and the cellulosic saccharification efficiency was evaluated in terms of final sugars recovery before fermentation.

Banana is one of the most important fruit crops in the world with an overall yearly production of more than 100 million tons of bananas, with a ratio of waste and product of 2:1. The rachis is the stalk of the inflorescence from the first fruit to the male bud. After fruit harvesting, banana lignocellulosic biomass (rachis, foliage, and stems) represent a waste and in producing countries as Ecuador or India the majority of the producers prefer to leave these residues to decompose outdoors, causing environmental problems such as the spread of diseases or polluting groundwater [34]. Some attempts have been done to use those materials for cellulose fiber recovery [35] or thermovalorization [36], but to the best of the Authors’ knowledge, no information is still available in scientific literature about chemical delignification treatments on banana rachis as a key step for bioethanol production. However, it could represent a great amount of valuable lignocellulosic materials, thus constituting an additional economical profit to farmers [37]. Considering the total amount of banana rachis produced in Ecuador per year, it has been estimated that 19 million liters of bioethanol could be produced [38].

## 2. Materials and Methods

### 2.1. Banana Rachis Samples

**Figure 1:**
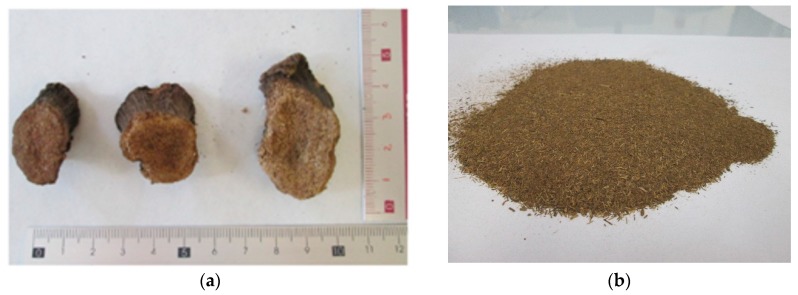
Samples of raw banana rachis (**a**) and rachis powder after grinding and drying (**b**).

The lignocellulosic residue was rachis from banana plants (*Musa paradisiaca* var. *barraganete*) cultivated and collected in the region of Guayas, Ecuador. 1000 g of samples were locally cut in small pieces and sent to our laboratory for subsequent treatments. The material was ground up, sieved using a <0.5 mm mesh size to obtain a homogeneous powder and then dried at 60 °C overnight (Figure 1). Sample was stored in a desiccator with CaCl_2_ at room temperature.

### 2.2. Chemical Pretreatments

#### 2.2.1. Acid-*organosolv* Pretreatment with Acetic Acid and Acetone (AA)

A mixture of glacial acetic acid (>99.85%), acetone (>99.5%) (Sigma Aldrich, Saint Louis, MO, USA) and water has been prepared at a ratio 10:50:40 (*v*/*v*) and pH adjusted to 2.7 with HCl 1M. Treatment was carried out on lignocellulosic substrate (20 g) by adding 250 mL of the acid-*organosolv* mixture for 30 min at boiling temperature under reflux. After treatment, the residual biomass was filtered on paper filter, extensively washed with water and buffered to neutral pH with NaOH 1M, and dried in a hot air oven at 60 °C overnight. The dried treated was weighed and stored in a desiccator with CaCl_2_. 

#### 2.2.2. Alcohol-*organosolv* Pretreatment with Ethanol (ET)

Rachis powder (20 g) was treated with 250 mL of 96% ethanol (>96.5%) (Sigma Aldrich) for 30 min at boiling temperature. The residual biomass was filtered on paper filter, extensively washed with water and dried in a hot air oven at 60 °C overnight. The dried treated was weighed and stored in a desiccator with CaCl_2_.

#### 2.2.3. Oxidative Pretreatment with Sodium Hypochlorite (SH)

A solution of NaClO 5% (250 mL) was added to rachis powder (20 g) at room temperature for 30 min. The residual biomass was filtered on paper filter and extensively washed with water and buffered to neutral pH with HCl 1M, and dried in a hot air oven at 60 °C overnight. The dried treated was weighed and stored in a desiccator with CaCl_2_.

#### 2.2.4. Oxidative Pretreatment with Electro Chemical Activated Solution Based on Hypochlorous Acid (ECA)

Rachis powder (20 g) was treated with Electro Chemical Activated solution based on Hypochlorous Acid (ECA)at pH 6 (250 mL) for 10 min at room temperature. According to Tamburini et al. [39], ECA solution was prepared by electrolysis of a solution of NaCl (5 g/L) in a flow-through electrochemical cell at pH 6 to allow hypochlorite/hypochlorous acid conversion. As a result, an ECA solution of about 1500 ppm of oxidizing substances (determined by iodometric titration and expressed as “active chlorine”) were obtained. Such solution, stored in glass containers and preferably in the dark, maintain its properties for some days. After treatment, the residual biomass was filtered on paper filter and extensively washed with water and buffered to neutral pH with NaOH 1M, and dried in a hot air oven at 60 °C overnight. The dried treated was weighed and stored in a desiccator with CaCl_2_.

#### 2.2.5. Oxidative Pretreatment with Hydrogen Peroxide and Hydrogen Peroxide with Alkali (HP and HPA)

To rachis powder (20 g) H_2_O_2_ 2% (*v/v*) (900 mL) was added (sample hydrogen peroxide, HP). In sample hydrogen peroxide with alkali (HPA) NaOH solution 5% (*w*/*w*) (100 mL) was added alongside. The final volume of HP and HPA was completed up to 1000 mL with water. Both treatments were kept under stirring for 90 min at room temperature. The the residual biomass was vacuum filtered in glass microfiber, washed with water until the hydrogen peroxide was completely removed, buffered to neutral pH with HCl 1M and dried in a hot air oven at 60 °C overnight. The dried treated was weighed and stored in a desiccator with CaCl_2_.

#### 2.2.6. Combination of Electro Chemical Activated solution based on Hypochlorous Acid and Ethanol (ECA + ET)

To rachis powder (40 g) 96% ethanol (500 mL) was added and refluxed for 30 min. The solid suspension was separated by filtration and dried at 60 °C overnight. The dried powder (20 g) was thereafter mixed up with ECA solution (250 mL) for 10 min at room temperature. After filtration the powder was dried at 60 °C up to constant weight, weighed and stored in a desiccator with CaCl_2_.

#### 2.2.7. Combination of Electro Chemical Activated Solution Based on Hypochlorous Acid and Acetic acid and Acetone (ECA + AA) 

To rachis powder (40 g) a mixture of glacial acetic acid:acetone:water (10:50:40) (500 mL) was added and refluxed for 30 min. The solid suspension was separated by filtration and dried at 60 °C overnight. The treated powder (20 g) was thereafter put in contact with ECA solution (250 mL) for 10 min at room temperature. After filtration the powder was dried at 60 °C up to constant weight, weighed and stored in a desiccator with CaCl_2_.

### 2.3. Hydrolysis of Pretreated Samples

Delignificated rachis samples have been submitted to enzymatic hydrolysis. 50 μL of cellulase from *Trichoderma reesei* (Novozyme 2730, ≥700 unit/gram of enzyme), supplemented with 50 μL of beta-glucosidase from *Aspergillus niger* (Novozyme 2605, ≥1000 U/g) were used for saccharifying the cellulosic material. Enzymatic hydrolysis has been carried out on 1 g of sample in 50 mM citrate phosphate buffer (pH 5.0). The substrate with buffer was pre-incubated at 50 °C on a rotatory shaker (Innova-40, NewBrunswick Scientific, Nürtingen, Germany) at 150 rpm for 2 h. 0.3 g of Tween 80 (1%, *v*/*v*) was also added to the reaction mixture and the reaction continued up to 72 h [40]. Samples of enzymatic hydrolysate were analysed for amount of monosaccharides released.

### 2.4. Analytical Methods

The chemical composition (cellulose, lignin, emicellulose, moisture and ash) of untreated rachis sample and of all the residual solid fraction post pretreatments were determined following standard Technical Association of Pulp and Paper Industry (TAPPI) protocols [41]. The monosaccharides (glucose, xylose and arabinose) released after hydrolysis were quantitatively estimated using High Performance Liquid Chromatography (HPLC) with a refractive index detector (Jasco RI-4030, Easton, MD, USA). A Rezex ROA-Organic Acid H^+^ (8%), 300 × 7.8 mm (Phenomenex, Torrance, CA, USA) was used at 80 °C. Isocratic elution was carried out with H_2_SO_4_ 0.01M water at 0.6 mL/min. Samples were analyze in triplicate. Infrared spectroscopic analysis of residual lignin has been carried out by means of Fourier transform infrared (FT-IR) spectrophotometer (Perkin Elmer Spectrum 1000), using a KBr disc containing 1% finely ground samples. Through the Spectrum 10^®^ software, supplied with the instrument, the corresponding absorbance spectra were collected within the range of 4400 cm^−1^ to 500 cm^−1^, and processed for baseline correction, noise correction (smooth) and identification of the wave numbers of the main peaks. 

### 2.5. Statistics

All pretreatments trials have been carried out in triplicate and the results reported as average. Linearity was evaluated by linear regression analysis, which was used to calculate the correlation coefficient, *y*-intercept and slope of the regression line. The curves were validated by means of the analysis of variance (ANOVA).

## 3. Results and Discussion

### 3.1. Chemical Characterization of Untreated Banana Rachis

Banana rachis pretreated materials was the substrate used in this study. As previously reported in the Materials and Methods section, the raw material was analyzed to determine the chemical composition. In the best of the Authors’ knowledge, in literature compositional analysis of banana rachis has been reported only from Guerrero et al. [37], otherwise data have to be compared with chemical composition of date palm rachis, the most similar matrix [42]. The chemical characterization of our Ecuadorian banana rachis has revealed the presence of cellulose (36.5%), hemicellulose (22.3%), lignin (26.2%), and ash (15.2%). Comparing the lignin content of date palm rachis has been found in the range 14–27%, and cellulose in the range 30–44%, whereas banana rachis analyzed in the cited paper has a content of lignin, cellulose, and hemicellulose of 10.8%, 26.1%, and 11.2%, respectively. All values are expressed on the basis of dry weight. These data led us to the conclusion that the chemical composition of rachis depends more up to region of cultivation (Ecuador versus Morocco for date palm and Spain for banana) than to the cultivar or species. The high cellulose content of Ecuadorian banana rachis qualifies it as potential valuable agricultural biomass for bioenergy production.

### 3.2. Delignification Efficiency of Chemical Pretreatments

To facilitate a general view of the different conditions applied during chemical treatments, a synoptic table is proposed (Table 1).

The different combinations of times and temperatures of treatments resulted from a series of trial-and-error experiments in our laboratory with the aim to maximize delignification yield for each treatment. Notably, taking the amount of raw material as starting invariant, conditions were very different, and unavoidably had different impacts, in terms of costs and in terms of environmental burden. Even though a detailed description of both of those aspects are beyond the aim of this study, a balance between efficiency and energy/water consumption should be done when defining the best treatment. 

**Figure 2:**
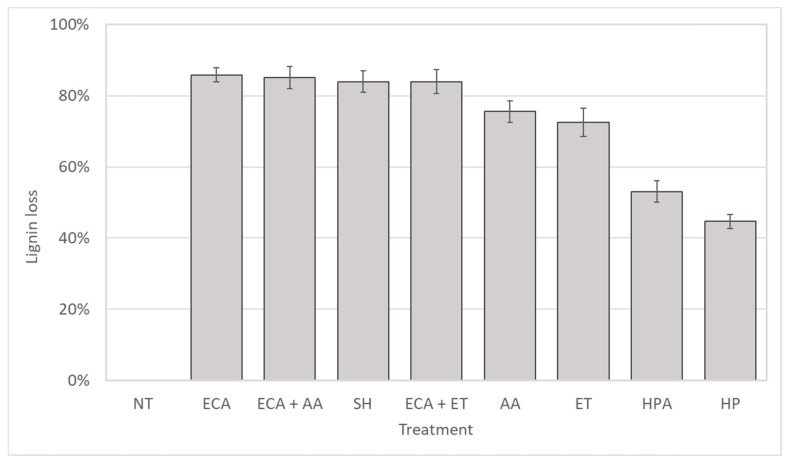
Lignin loss (% *w*/*w*) expressed as percentage of lignin removal in comparison with lignin content before sample pretreatments. NT: not treated; ECA: Hypochlorous acid; ECA+AA: Hypochlorous acid + Acid-*organosolv*; SH: Sodium Hypochlorite ECA + ET: Hypochlorous acid + Alcohol-*organosolv;* AA: Acid-*organosolv*; ET: Alcohol-*organosolv*; HPA: Hydrogen Peroxide Alkaline; HP: Hydrogen Peroxide.

Effects of different chemical pretreatments on rachis powder, compared to not-treated sample, are shown in Figure 2. Treatments based on active chlorine (sodium hypochloride (SH), ECA, ECA + AA, and ECA + ET) were more effective than treatments based on hydrogen peroxide, which both (HP and HPA) leaved great amounts of residual lignin in the samples. Even though among chemical treatments the *organosolv* are the most commonly used methods to pretreat lignocellulosic biomass, they have proved not to be so successful for banana rachis. Reporting data in terms of percentage of lignin loss, i.e., delignification capacity of various treatments, compared with 0% of not-treated sample (NT), it is even more evident that treatments based on the oxidant effect of chlorine show high and comparable efficiency.

The results indicate that the pretreatment of biomass with oxidizing ECA solution combined with both *organosolv* does not produce a significant advantage over the absolute yield of the dignified biomass treated only with the oxidizing ECA solution. The oxidizing treatment with the ECA solution simultaneously removes a similar amount of soluble substances and lignin, without adding solvents. 

Even SH pretreatment has permitted to reach good lignin losses, comparable to ECA solution, alone or in combination with *organosolv*. Anyway, in a perspective of practical use, it is worthwhile pointing out that sodium hypochlorite is a chemical reagent that needs to be removed from the solution at the end of the reaction, hypochlorous acid naturally decades to chloride after 48–72 h (experimental evidence), reducing the overall environmental impact of the treatment.

**Figure 3:**
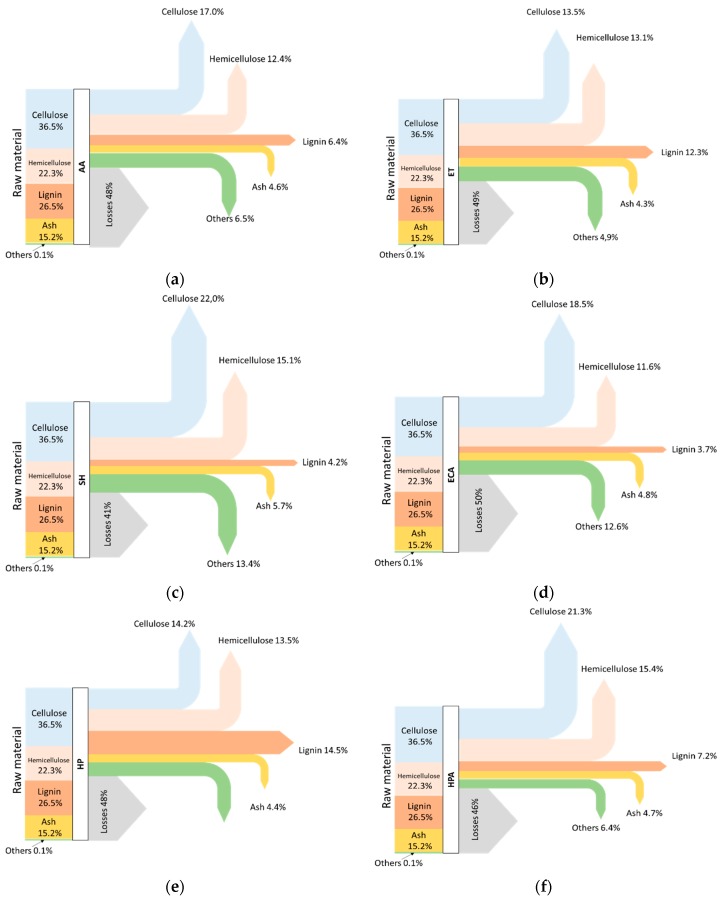

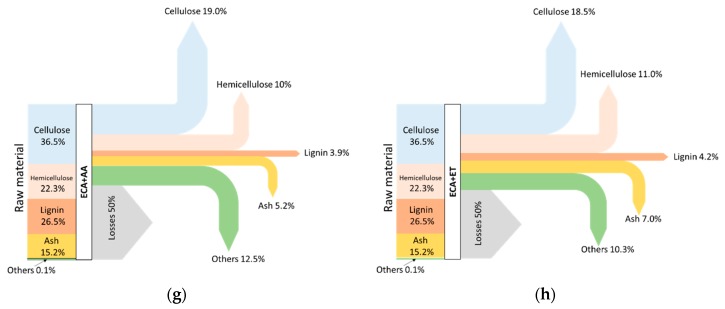
Mass balance after delignification treatment AA (**a**); ET (**b**); SH (**c**); ECA (**d**); HP (**e**); HPA (**f**); ECA + AA (**g**) and ECA + ET (**h**).

A mass balance for each treatment has been carried out based on the chemical characterization of samples before hydrolysis (Figure 3). 

Measuring mass of the samples before and after the delignification treatments allowed for calculation of the weight loss caused by the delignification. As expected, all treatments lead to material losses in the range 40–50% [43]. Others have been calculated as difference and represent the not-characterized fraction that includes part of the compounds deriving from lignocellulose degradation. 

### 3.3. Enzymatic Saccharification

The results of enzymatic hydrolysis and quantification of monosaccharides recovery are shown in Table 2. It has been reported that a hydrolysis of pretreated biomass can be considered valuable when it gives a concentration of at least 10% of free glucose in the medium for later fermentation [44], so all treatments have given satisfying results. Besides glucose, xylose, and arabinose were analyzed because they usually can be found after hydrolysis being the principal components of hemicellulose [45] fraction. Hemicellulose could be partially hydrolyzed during delignification treatments. 

The presence of small amount of free monosaccharides in not-treated rachis sample could derive from natural phenomena, and they have been quantified as a benchmark. The overall amount of free pentoses (xylose and arabinose) were low, and their concentration in some cases decrease after treatments, compared with NT sample, probably due to degradation occurring in the severe conditions maintained during chemical delignification. The enzymatic saccharification of all pretreated samples showed a significant conversion of cellulose to glucose because of lignin and/or hemicellulose removal during pretreatment had permitted to cellulase and beta-glucosidase to act on their elective substrate (i.e., cellulose). It is worthwhile noting that, in general, glucose recovery followed the same trend of delignification, namely higher is the lignin loss higher is the enzymes efficiency of hydrolyze cellulose. From one side it is due to the barrier disruption which “unlocks” cellulose to enzymes, from the other side the limited enzymatic saccharification in the presence of higher lignin content can be due to the high affinity of cellulase towards lignin, which resulted in unavailability of enzyme to cellulose moieties and led to poor saccharification yields. Similar observation have been reported by other Authors [46,47].

Among the different samples evaluated here, those treated with the active chlorine were observed to be more vulnerable to enzymatic hydrolysis and resulted in maximum glucose recovery. The higher enzymatic saccharification in SH, ECA, ECA + AA, and ECA + ET pretreated samples can be attributed to the presence of higher free cellulose content with a minimum amount of residual lignin (3.7–4.2%). On the other hand, the hydrogen peroxide treatments with minimum lignin removal showed higher glucose recovery than both *organosolv* treatments. This observed reverse correspondence between percentage of delignification and glucose recovery in the case of *organosolv* (AA and ET) and hydrogen peroxide (HP and HPA) treatments can be right explained by different actions of various chemical agents on lignin structure [48]. It has been reported by several workers that delignification not only remove the lignin but also act as a swelling agent, which in turn enhances the surface area of the sample and make the cellulose more amenable for enzymatic action [49].

**Figure 4:**
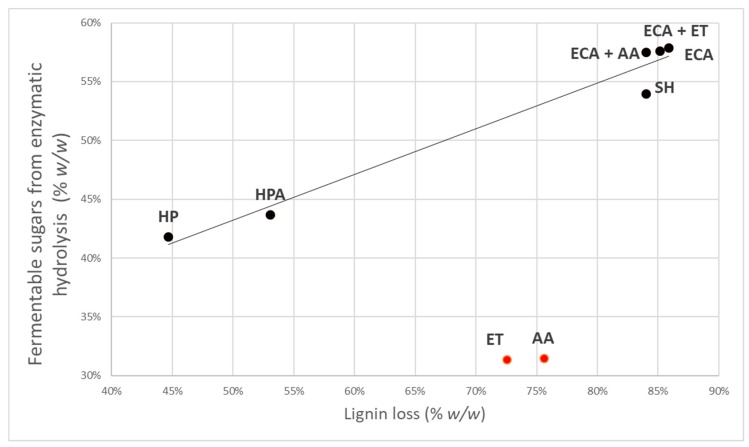
Relation between fermentable sugars from enzymatic hydrolysis and lignin loss. The solid line represents the linear correlation (R^2^ = 0.9) between enzymatic digestibility and delignification yield for all samples. The black points have been included in the linear model; red points are outliers according to Mahalanobis distance criterion.

Plotting the obtained results of enzymatic hydrolysis and rachis delignification yields, linear relation can be observed (R^2^ > 0.97; *p* = 0.05) for all treatments, except for *organosolv*-treated samples that resulted as outliers (Figure 4). The analytical data have been validated by means of ANOVA that demonstrated significant linear regression (F calculated = 125.64 > F critical = 3.78; P = 5%). 

These data show that delignification yield has a great influence on sugars recovery from enzymatic hydrolysis, confirming data obtained by Toscan et al. [50], where correlation with R^2^ > 0.9 have been reported. This seems to support the statement that lignin is a relevant inhibitor of enzymatic hydrolysis not only for structural hindrance, but also for the irreversible absorption of cellulase on lignin itself, reducing the effective availability of active enzyme for hydrolysis. The plot also confirms that in the condition maintained in these experiments, both *organosolv* treatments gave a particularly low and anomalous sugars recovery after hydrolysis. 

### 3.4. Comparison of Pre-Treatments Effect on Lignocellulose Structure by Fourier Transform Infrared

The accessible surface area is one of the key factors affecting the hydrolysis of lignocellulosic material and the overall process efficiency. Pore dimensions, bond cleavages, and structural breakage induced by the chemical agents have a great influence on enzymatic attack efficiency [51].

As demonstrated elsewhere [52], different chemical treatments based on ECA solution, sodium hypochlorite, and *organosolv* gave rise to different residual lignocellulose structure. ECA solution was the most efficient in lignin degradation, whereas sodium hypochlorite and *organosolv* seemed to have more effect against inter-and intra-polymeric bonds of other polysaccharides of the lignocellulose structure. This finding is supported by several other authors [53,54] who suggested that the main wood component determining enzymatic hydrolyzability is not only the amount of lignin itself, but also its post-delignification structure and organization within the other wood cell wall components. In fact, beyond the quantitative aspects of the delignification yield, what differs considerably in the different treatments is the type of fragments that are produced during the action of the delignificant agents. During the process, the fragmenting and flaking of polymers induce a partial breakdown of the chemical structure and subsequently lead to an increase of internal surface area and median pore volume. 

**Figure 5:**
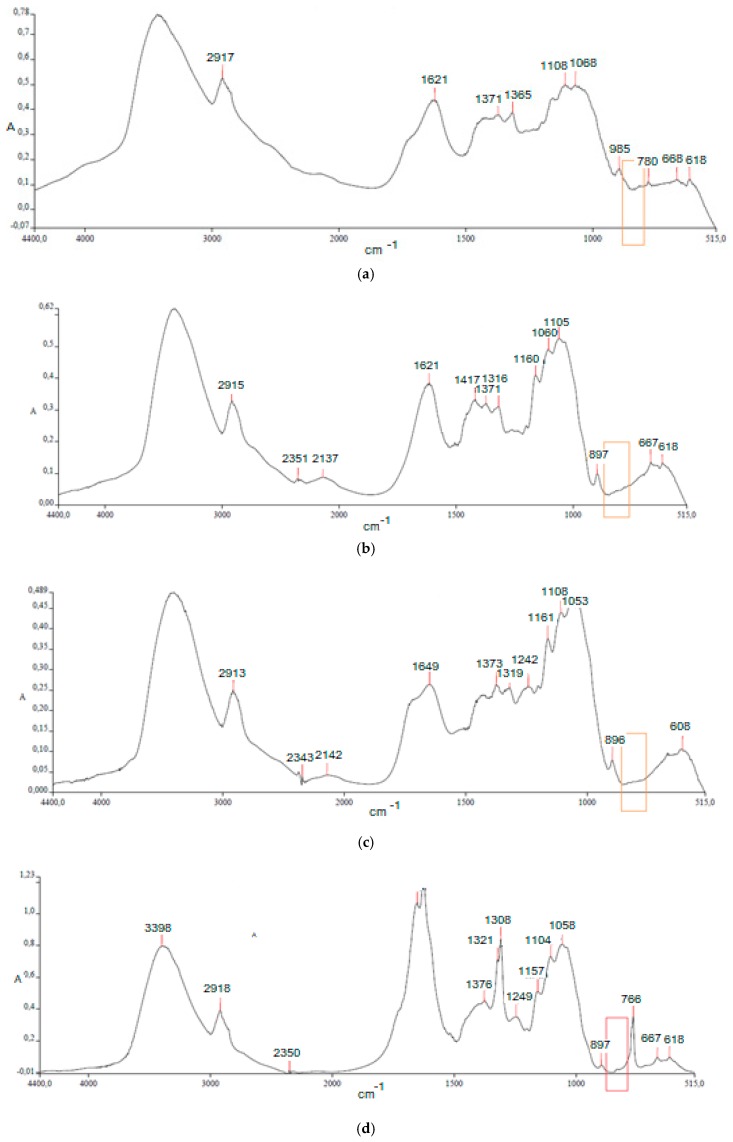
FT-IR spectra of rachis samples not-treated (**a**), treated with ECA solution (**b**), with *organosolv* AA solution (**c**), and with hydrogen peroxide alkaline (**d**), in the range 4400–515 cm^−1^.

Non-destructive elucidation and analysis of the lignin and hemicellulosic samples are nowadays carried out primarily by spectroscopic methods, which constitute important tools in connection with the characterization of the polymers [55]. In this study, we focused on Fourier transform infrared (FT IR), which has enabled structural information to be derived from the intact residual lignocellulose, avoiding the possibility of degradation artifacts. Figure 5 illustrated the FTIR spectra of not-treated rachis sample, compared with FTIR of samples treated with active chlorine (ECA), *organosolv* (AA), and hydrogen peroxide (HP), as examples of the paradigm of chemical pretreatments here applied.

In the spectrum of not-treated samples (Figure 5a), typical peaks corresponding to lignin, cellulose and hemicellulose can be recognized. In particular, signals at 3600–3000 cm^−1^ corresponding to OH-stretching and at 2900–2800 cm^−1^ corresponding to CH_n_ stretching, together with signals at 1170–1100 and 1060 cm^−1^, corresponding to C–O–C stretching of pyranose ring skeletal and C–OH alcoholic bonds of sugars, are found in FTIR spectra of pure cellulose and hemicellulose [56]. Lignin is mainly responsible of signals in the finger print region (1830–700 cm^−1^). This group of complex and superimposed peaks could indicate rachis lignin rich of metoxyl–O–CH_3_, C–O–C aryl-alkyl ether linkages containing compounds and C=C bonds from aromatic rings (corresponding to 1700, 1371–1316, and 1621 cm^−1^, respectively).

Oxidant treatment with ECA solution (Figure 5b) seems to have the most dramatic effect on lignin structure, disappearing the peak at 1730 cm^−1^ of C=O stretching of carbonyl bonds and comparing weak signals at 1500 and 1250 cm^−1^, probably due to degradation fragments. Moreover, two new peaks are evidenced at 2351 and 2137 cm^−1^, probably due to the production of nitrogen-based degradation products. During the oxidative delignification process, oxidation reagents release a large number of free radicals, resulting in remarkable oxidative fragmentation and removal of lignin from lignocellulosic matrix. 

According to literature [57], IR-spectra analysis confirms that with the assistance of oxidation reagents, almost all lignin can be removed from lignocellulosic materials with the remaining of most cellulose and hemicelluloses, permitting a higher recovery of fermentable sugars. In the spectrum of rachis treated with *organosolv* AA (Figure 5c), on the contrary, the degrading effect on lignin appears less effective, corresponding to the low delignification yield observed here.

The oxidative action of hydrogen peroxide-derived radicals is thought to contribute to the depolymerization of lignin by attacking lignin side chains and fragmenting lignin into a number of low molecular weight compounds [58]. In fact, the fingerprint region shows a very different profile, with two spiky peaks at 1650 and 1308 cm^−1^ (Figure 5d). It is anyway worthwhile noting as peaks corresponding to cellulose and hemicellulose are here less intense, being due to a possible degradation during treatment, which reflects the low cellulose-to-glucose conversion during hydrolysis. 

## 4. Conclusions

Delignification and saccharification of banana rachis for bioethanol production were performed and evaluated. Pretreatments have been chosen to compare solvolitic effects (*organosolv*) and oxidant effects with active chlorine in form of hypochlorite/hypochlorous acid and hydrogen peroxide, with and without alkali. Hypochlorous acid was the highest performing in terms of delignification yield, and its combinations with *organosolv* did not reach a significant improvement. In general, active chlorine oxidations were the most effective for lignin removal, meanwhile obtaining the most promising cellulose-to-glucose conversion. Further research will be focused on optimizing the ECA pretreatment up to be applied as a valuable industrial delignification process. 

## Figures and Tables

**Table 1:** Comparison among different chemical treatments.

#	Pretreatment	Chemical Agent	Temperature (°C)	Time (min)	pH
1	Acid-*organosolv* (AA)	glacial acetic acid:acetone:water (10:50:40)	100	30	2.7
2	Alcohol-*organosolv* (ET)	96% Ethanol	100	30	-
3	Sodium Hypochlorite (SH)	NaClO 5%	25	30	11
4	Hypochlorous acid (ECA)	HClO/ClO^−^	25	10	6
5	Hydrogen Peroxide (HP)	H_2_O_2_ 2%	25	90	-
6	Hydrogen Peroxide Alkaline (HPA)	H_2_O_2_ 2% + NaOH 5%	25	90	14
7	Hypochlorous acid + Acid-*organosolv* (ECA+AA)	Treat.#4 + Treat.#1			
8	Hypochlorous acid + Alcohol-*organosolv* (ECA+ET)	Treat.#4 + Treat.#2			

**Table 2:** Fermentable sugars recovery after enzymatic hydrolysis of pretreated banana rachis samples (NT = Not-treated).

Pretreatment	Glucose (% *w*/*w*)	Xylose (% *w*/*w*)	Arabinose (% *w*/*w*)
Rachis (NT)	2.8 ± 0.2	9.0 ± 0.7	1.5 ± 0.0
Acid-*organosolv* (AA)	16.6 ± 0.7	12.8 ± 1.0	2.1 ± 0.0
Alcohol-*organosolv* (ET)	19.8 ± 0.6	10.8 ± 0.7	0.8 ± 0.0
Sodium Hypochlorite (SH)	44.7 ± 1.1	5.0 ± 0.3	1.8 ± 0.0
Hypochlorous acid (ECA)	51.4 ± 1.2	4.2 ± 0.2	2.3 ± 0.0
Hydrogen Peroxide (HP)	35.1 ± 1.8	5.2 ± 0.3	1.5 ± 0.0
Hydrogen Peroxide Alkaline (HPA)	36.5 ± 1.5	5.6 ± 0.3	1.6 ± 0.0
Hypochlorous acid + Acid-*organosolv* (ECA + AA)	48.3 ± 1.4	7.5 ± 0.4	1.8 ± 0.0
Hypochlorous acid + Alcohol-*organosolv* (ECA + ET)	48.7 ± 1.4	6.9 ± 0.3	1.9 ± 0.0
